# Adaptation of Rhizosphere Microbial Communities to Continuous Exposure to Multiple Residual Antibiotics in Vegetable Farms

**DOI:** 10.3390/ijerph20043137

**Published:** 2023-02-10

**Authors:** Jincai Qiu, Yongshan Chen, Ying Feng, Xiaofeng Li, Jinghua Xu, Jinping Jiang

**Affiliations:** 1 School of Advanced Manufacturing, Fuzhou University, Quanzhou 362000, China; 2 School of Resources and Environmental Science, Quanzhou Normal University, Quanzhou 362000, China; 3 Guangxi Collaborative Innovation Center for Water Pollution Control and Water Safety in Karst Area, Guilin University of Technology, Guilin 541004, China

**Keywords:** vegetable farms, antibiotics, rhizosphere, microbial community, organic fertilizers, canonical-correlation analyses

## Abstract

The constant application of manure-based fertilizers in vegetable farms leads to antibiotic residue accumulation in soils, which has become a major stressor affecting agroecosystem stability. The present study investigated the adaptation profiles of rhizosphere microbial communities in different vegetable farms to multiple residual antibiotics. Multiple antibiotics, including trimethoprim, sulfonamides, quinolones, tetracyclines, macrolides, lincomycins, and chloramphenicols, were detected in the vegetable farms; the dominant antibiotic (trimethoprim) had a maximum concentration of 36.7 ng/g. Quinolones and tetracyclines were the most prevalent antibiotics in the vegetable farms. The five most abundant phyla in soil samples were *Proteobacteria*, *Actinobacteria*, *Acidobacteria*, *Chloroflexi* and *Firmicutes*, while the five most abundant phyla in root samples were *Proteobacteria*, *Actinobacteria*, *Bacteroidetes*, *Firmicutes* and *Myxococcota*. Macrolides were significantly correlated with microbial community composition changes in soil samples, while sulfonamides were significantly correlated with microbial community composition changes in root samples. Soil properties (total carbon and nitrogen contents and pH) influenced the shifts in microbial communities in rhizosphere soils and roots. This study provides evidence that low residual antibiotic levels in vegetable farms can shift microbial community structures, potentially affecting agroecosystem stability. However, the degree to which the shift occurs could be regulated by environmental factors, such as soil nutrient conditions.

## 1. Introduction

Fertilization using livestock and poultry manure is considered a primary method through which antibiotics enter the soil environment [1,2]. Continuous application of manure or manure-based fertilizers leads to the frequent detection of antibiotics and their resistant genes in farmland ecosystems, which has become an environmental health and food chain safety concern [3,4]. The use of manure on farmland considerably increases the levels of antibiotic residues and expression of antibiotic resistant genes, thereby posing a public health risk through the migration and diffusion of antibiotics and their resistance genes in the soil environment [5,6,7,8]. Antibiotics can affect microbial communities by altering the dominant flora, community composition and structure, as well as microbial diversity and richness; however, the magnitude of the effects is substantially moderated by various environmental factors [9,10,11,12]. Soil physiochemical properties, such as organic matter, nutrient availability, and pH, play a key role in shaping microbial communities, and consequently, affect the ability of agroecosystems to adapt to antibiotic residues [13,14]; therefore, the shifts in microbial communities exposed to antibiotics are the result of the interaction between antibiotics and environmental factors.

Laboratory experiments have shown that the antibiotic activity of a microbial community is influenced by the concentration, exposure time, and type of antibiotic [15]. Antibiotic effects can be attributed to single species such as *Aeromonashydrophila*, *Aeromonassalmonicida*, and *Ediwardsiellatarda*, as well as larger microbial communities such as fungi and bacteria [15,16]. Observations have shown that the percentages of some classes, including *Bacilli*, *Bacteroidia* and *Gammaproteobacteria*, have significantly grown in experiments involving veterinary antibiotics exposure, which are known to contain bacteria with tolerance and degradation capabilities [16,17,18]. *Proteobacteria* and *Bacteroidetes*, both of which are known to contain antibiotic resistance genes, are often seen to be forming communities that are resistant to antibiotics after a prolonged period of antibiotic usage [19,20]. However, agricultural farms receive multiple antibiotics during fertilization, and their residual concentrations in soils are much lower than those used in laboratory experiments. Low concentrations of antibiotics originating from external sources may not necessarily promote microbial diversity and functional stability in actual environments, especially over the long term for microbes that have already developed antibiotic resistance [9,21]. Co-resistance profiles between antibiotics and environmental stressors, such as heavy metals, may mask the effects of antibiotic residues on microbial communities because metals and metalloids existed earlier and their concentrations may be above the permissible limits for soil microorganisms causing significant anthropogenic emissions [13,22,23]. Environmental conditions, such as temperature, pH, redox potential, metals, and nutrients, can regulate microbial abundance and population structure, thereby promoting the effects of antibiotics on microbial communities. Therefore, microbial community (structure and function) responses to exposure to residual antibiotics at levels that are detected in the actual environment are complex and require further exploration.

The rhizosphere is a sensitive soil area that is susceptible to exogenous chemicals, where roots interact with physical, chemical, and biological soil properties [24]. Several studies have shown that manure application substantially increases the antibiotic resistance profiles of microbial communities in the rhizosphere, root endophytes and phyllosphere of crops, such as corn, wheat, rice, and vegetables [25,26,27]. However, microbiomes with antibiotic resistance profiles are determined by a combination of indigenous microbes and antibiotic residuals, and are regulated by a variety of environmental factors [28,29]. Therefore, evaluation of microbial community responses to exposure to residual antibiotics must comprehensively consider the interrelationships among antibiotics, environmental factors, and indigenous microbes. In this study, we characterized the microbial community (diversity and dominant microbes) in the vegetable rhizosphere of a suburban vegetable growing area in Jinjing City, Fujian Province, China, where manure-based fertilizer has been used for several years. Our objective was to characterize in situ changes in the microbial community in the rhizosphere microenvironment comprising soils and roots, which are caused by exposure to multiple antibiotic compounds at actual residual concentrations. We hypothesized that there would be a shift in the microbial community between rhizosphere soil and roots due to the residual antibiotics (type and dosage) associated with various farming practices.

## 2. Materials and Methods

### 2.1. Chemicals and Standards

All standard chemicals used for analyses were purchased from Dr. Ehrenstorfer GmbH (Augsburg, Germany), except for the internal standards (erythromycin-13C-d3, lincomycin-d3, sulfamethoxazole-d4, ciprofloxacin-d8, doxycycline-d3, and chloramphenicol-d5), which were purchased from Sigma-Aldrich (St. Louis, MO, USA). High performance liquid chromatography (HPLC)-grade solvents, including methanol, formic acid, and acetonitrile, were provided by Merck & Co., Inc. (Kenilworth, NJ, USA). Guaranteed reagents, including ammonium acetate, hydrochloric acid, sodium hydroxide, dipotassium hydrogen phosphate, and disodium ethylenediaminetetraacetic acid (EDTA), were purchased from Sinopharm Chemical Reagent Co., Ltd. (Shanghai, China). Ultrapure water was prepared using a water purification system. The internal standards were dissolved in acetonitrile as stock solutions and stored in a freezer at −40 °C. Working standard solutions were prepared immediately before measurements were taken by diluting the stock solutions with a mixture of menthol and water (9:1, *v*/*v*). For solid-phase extraction (SPE), Oasis HLB cartridges (6 mL/500 mg) were purchased from Water Oasis Co., Ltd. (Milford, MA, USA), glass microfiber filters (Whatman GF/F) were obtained from Sigma-Aldrich (St. Louis, MO, USA), and syringe-driven filters (PTFE) from Millipore Corp. (Bedford, MA, USA).

### 2.2. Site Selection and Rhizosphere Sample Collection

Rhizosphere samples were collected in December 2021 from vegetable farms in the city of Jinjiang, Fujian Province, China. The suburban vegetable fields are vegetable basket projects that have been in operation for nearly 10 years. Livestock or poultry manure is often used as a basal fertilizer in each growing season before planting vegetables. Eleven types of vegetable farms, including *Brassica chinensis* (V1), *Spinacia oleracea* (V2), *Lactuca sativa* var. *ramosa Hort.* (V3), *Brassica juncea* (V4), *Brassica narinosa* L. (V5), *Brassica campestris* (V6), *Brassica pekinensis* (V7), *Lactuca sativa* var. *angustana* (V8), *Capsella bursa-pastoris* (V9), *Coriandrum sativum* (V10), and *Apium graveolens* (V11) were selected for rhizosphere samples of soils and plants. Rhizosphere soils from ten sites, randomly distributed in each vegetable farm, were gathered employing the method described by Gremion et al. [30] and then blended completely to generate a composite sample. Briefly, the plants and soil from the fields were taken away, and each plant was gently shaken to remove the major part of the soil. The soil still attached to the roots was then referred to as rhizosphere soil. The sample was divided from the roots by stirring it in 50 mL of sterile 0.9% NaCl solution for 5 min, followed by centrifugation at 8000× *g* for 10 min. Whole plants collected from the 10 sampled sites in each vegetable farm were immediately placed in fresh-keeping boxes maintained at 4 °C and transported to the laboratory for processing.

The soil samples were stored in a freezer at −40 °C for a week prior to lyophilization using a freeze-dryer (FD-1C-50; Boyikang Laboratory Instruments Co., Ltd., Beijing, China). The dried samples were ground and sieved, with 0.15 mm being used for analyses of antibiotics and heavy metals, and 2.00 mm for analyses of pH and soil nutrient conditions.

The vegetable roots were treated as follows: roots were rinsed under running water for 3 min and excess water was removed with sterile absorbent papers. Root materials were then sterilized by immersion in 30% hydrogen peroxide for 30 min, followed by rinsing in sterile Milli-Q water (Millipore Corp., Bedford, MA, USA), and washed with 70% ethanol for 1 min. The treated roots were then stored in a freezer at −40 °C for subsequent DNA extraction.

### 2.3. DNA Extraction, PCR Amplification, and Illumina High-Throughput Sequencing

Genomic DNA of the rhizosphere soil and the disinfected leaves and roots were extracted using the E.Z.N.A. soil DNA extraction kit (Omega Bio-tek, Norcross, GA, USA), according to the manufacturer’s protocols. The V4–V5 region of the bacterial 16S ribosomal RNA gene was amplified by polymerase chain reaction (PCR) (95 °C for 2 min, followed by 25 cycles at 95 °C for 30 s, 55 °C for 30 s, and 72 °C for 30 s, and a final extension at 72 °C for 5 min) using 515F (5′-barcode- GTGCCAGCMGCCGCGG)-3′ and 907R (5′-CCGTCAATTCMTTTRAGTTT-3′) primers for soil samples and 799F (5′-A-ACMGGATTAGATACCCKG-3′) and 1193R (5′-ACGTCATCCCCACCTTCC-3′) primers for root samples, where the barcode used was an eight-base sequence unique to each sample. The PCR reactions were performed in triplicate in a 20 μL mixture containing 4 μL of 5 × FastPfu Buffer, 2 μL of 2.5 mmol/L dNTPs, 0.8 μL of each primer (5 μmol/L), 0.4 μL of FastPfu Polymerase, and 10 ng of template DNA. The PCR products were detected on 2% agarose gels and purified using an AxyPrep DNA gel extraction kit (Axygen Biosciences, Union City, CA, USA), according to the manufacturer’s instructions.

Purified PCR products were quantified using a Qubit 3.0 fluorometer (Life Technologies, Carlsbad, CA, USA) and every 24 amplicons with different barcodes were mixed equally. The pooled DNA product was used to construct the Illumina paired-end library following the Illumina genomic DNA library preparation procedure. The amplicon library was paired-end sequenced (2 × 250 bp) on an Illumina MiSeq platform (Illumina, San Diego, CA, USA), according to standard protocols. The quality of the reads was verified using QIIME (version 1.17), and chimeric reads were removed using UCHIME (http://www.drive5.com/usearch/manual/uchime_algo.html, accessed on 7 September 2022). Finally, the sequences were clustered into operational taxonomic units (OTUs) based on the 97% similarity cutoff using UPARSE (version 7.1, http://drive5.com/uparse/, accessed on 7 September 2022), matched with the 16S rRNA sequence database, and used for further analyses.

### 2.4. Soil Antibiotics Extraction and Quantification

Soil samples were subjected to ultrasonic-assisted extraction (USE) coupled with SPE, as described by Huang et al. (2013) [31]. Briefly, a mixture of 5 g soil sample in 25 mL extraction solvent consisting of an (EDTA-SPB with acetonitrile: Mg(NO_3_)_2_-NH_3_·H_2_O, *v*/*v*, 3:1) was transferred into a 50-mL glass centrifuge tube and kept in the dark overnight. Afterward, the mixture was extracted by USE for 30 min, centrifuged, and the supernatant was collected. The soil pellets were resuspended in 25 mL of extraction solvent, subjected to 30 min of ultrasonic extraction, and the supernatant was again collected by centrifugation. The extraction procedure was repeated one more time, and then three supernatants were combined and filtered through a glass microfiber filter (0.7 μm). The filtrates were further diluted to 500 mL using ultrapure water prior to hydrophilic–lipophilic balanced (HLB)-SPE. After the soil extract diluents were passed through the pre-conditioned SPE column at a flow rate of 3.0 mL/min, the extracted antibiotics were eluted with methanol and then concentrated to 1.0 mL by nitrogen gas prior to high performance liquid chromatography-tandem mass spectrometry.

Chromatographic separation was achieved using an HPLC system (Agilent 1290; Agilent Technologies, Santa Clara, CA, USA), which was equipped with a Waters Acquity UPLC HSS T3 column (2.1 mm × 100 mm, 1.8 μm) maintained at a constant temperature of 40 °C. Mobile phase A was 5 mM ammonium acetate and B was acetonitrile. The gradient elution program for the separation of antibiotics was as follows: 0 min, 10% B; 5 min, 15% B; 7 min, 20% B; 11 min, 40% B; 15 min, 60% B; and held at 90% B for 5 min. The total flow rate was 0.4 mL/min and the sample injection volume was 5 μL. A mass spectrometer (QTRAP 6500 PLUS System; AB SCIEX, Framingham, MA, USA) was used to detect and identify the targeted antibiotics. The precursor and product ions of each compound were analyzed in a multiple reaction monitoring system in the positive ion mode for target antibiotics (see Table S1 in the Supplementary Material).

### 2.5. Determination of Soil Physiochemical Properties

The soil pH was measured in a soil/deionized water slurry at a ratio of 1:2.5 using a pH-EC meter (Accumet Excel XL60; Fisher Scientific Inc., Hampton, NH, USA). The available phosphorus in soil samples was extracted using hydrochloric acid in ammonium fluoride and its content determined using molybdenum–antimony anti-colorimetry. Soil nitrate-nitrogen (NO_3_^−^-N) and ammonium-nitrogen (NH_4_^+^-N) were extracted using 0.01 mol/L anhydrous calcium chloride and quantified using a flow injection autoanalyzer. The soil total carbon content (TC), total nitrogen content (TN), and total sulfur content were measured using an elemental analyzer (Vario MAX CNS; Elementar Analysensysteme GmbH, Berlin, Germany). Heavy metals such as lead (Pb), chromium (Cr), zinc (Zn), nickel (Ni), copper (Cu), Cadmium (Cd) and arsenic (As) were digested by microwave-assisted acid digestion using trace-pure nitric acid (2.5 mL), hydrofluoric acid (1.5 mL), and a closed-vessel high-pressure microwave digester–Multiwave GO (Anton Paar, Graz, Austria), according to Chen et al. [32]. Finally, the metal concentrations were determined by inductively coupled plasma optical emission spectrometry (Optima 7000DV; PerkinElmer, Waltham, MA, USA). Physiochemical properties of sampled soils are presented in Table 1.

### 2.6. Statistical Analysis

Canonical correlation analysis (CCA) and redundancy analysis (RDA) were performed to explore the relationships between basic soil properties and residual antibiotics or microbial communities. Statistical analyses were conducted using SAS for Windows (version 9.2; SAS Institute Inc., Cary, NC, USA). The RDA was conducted using CANOCO (version 5.0; Microcomputer Power, Ithaca, NY, USA), and principal coordinate analysis (PCoA), heatmap, and correlation analyses were performed using R (version 3.4.1, https://cran.r-project.org/bin/windows/base/, accessed on 2 March 2023).

## 3. Results

### 3.1. Occurrence of Antibiotics in Rhizosphere Soil

**Figure 1:**
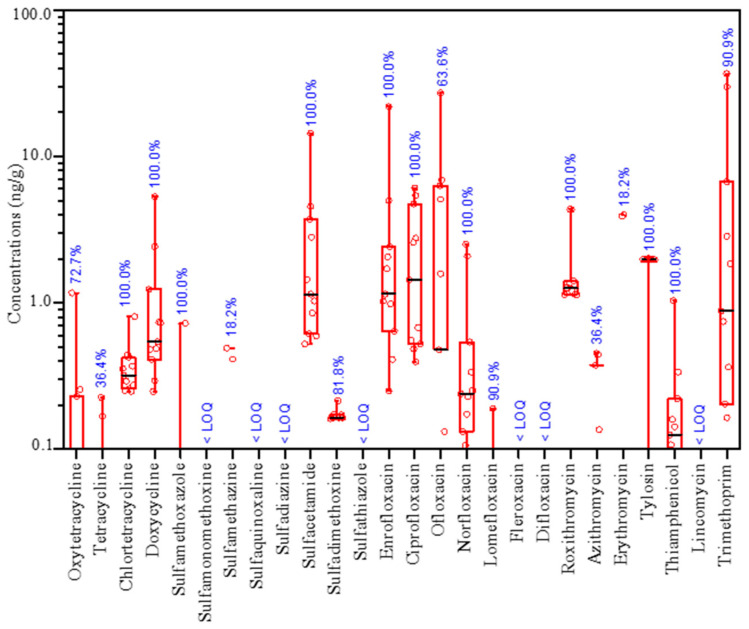
Occurrence of the selected antibiotics in rhizosphere soils. Note: the data beside the box diagram denote detection frequencies of antibiotics in 11 types of vegetable farms.

Quinolone and tetracycline antibiotics were the dominant contaminants in the samples analyzed, with high concentrations and detection frequencies being observed (Figure 1). Six of the seven quinolone antibiotics identified were present in the vegetable farms, with ofloxacin having the highest concentration of 27.1 ng/g in *B. pekinensis* farm; however, difloxacin was not detected in the samples. Chlortetracycline and doxycycline were present in all vegetable farms, with doxycycline having the highest concentration of 5.3 ng/g in the rhizosphere soil of *B. chinensis*. Five of the eight sulfonamide antibiotics were detected in all vegetable farms, with sulfacetamide having the highest concentration of 14.4 ng/g in the rhizosphere soil of *B. juncea*, whereas sulfamonomethoxine, sulfadiazine, and sulfathiazole were not detected in the samples. Roxithromycin and tylosin were detected in all samples, with roxithromycin having the highest concentration of 4.4 ng/g in the rhizosphere soil of *S. oleracea.* Based on individual compounds, trimethoprim was the most dominant antibiotic with the highest concentration (36.7 ng/g), followed by ofloxacin (27.1 ng/g), enrofloxacin (21.9 ng/g), and sulfacetamide (14.4 ng/g). The rhizosphere soil of *B. chinensis* had the highest concentration of the 26 targeted antibiotics (86.8 ng/g), followed by that of *S. oleracea* (54.7 ng/g), *B. pekinensis* (47.0 ng/g), and then *B. juncea* (28.1 ng/g).

### 3.2. Bacterial Community Structure in the Rhizosphere

**Figure 2:**
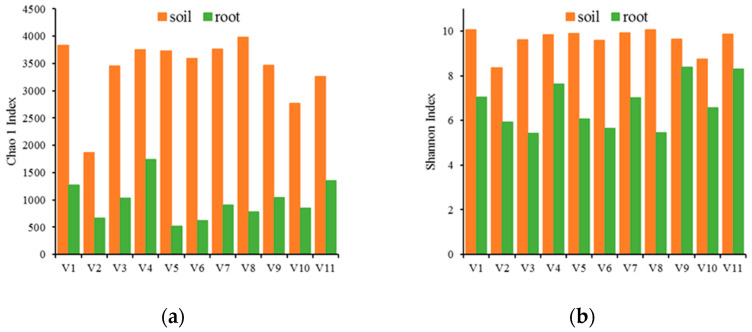
Microbial diversity in rhizosphere samples from different vegetable farms. (**a**) Chao 1 index; (**b**) Shannon index. *Brassica chinensis* (V1), *Spinacia oleracea* (V2), *Lactuca sativa* var. *ramosa Hort.* (V3), *Brassica juncea* (V4), *Brassica narinosa* L. (V5), *Brassica campestris* (V6), *Brassica pekinensis* (V7), *Lactuca sativa* var. *angustana* (V8), *Capsella bursa-pastoris* (V9), *Coriandrum sativum* (V10), and *Apium graveolens* (V11).

**Figure 3:**
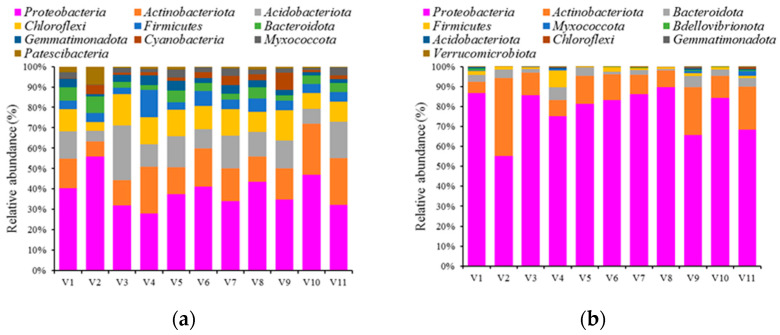
Relative abundances of bacterial phyla in rhizosphere samples from different vegetable farms. (**a**) soil samples; (**b**) root samples. *Brassica chinensis* (V1), *Spinacia oleracea* (V2), *Lactuca sativa* var. *ramosa Hort.* (V3), *Brassica juncea* (V4), *Brassica narinosa* L. (V5), *Brassica campestris* (V6), *Brassica pekinensis* (V7), *Lactuca sativa* var. *angustana* (V8), *Capsella bursa-pastoris* (V9), *Coriandrum sativum* (V10), and *Apium graveolens* (V11).

The variability in microbial communities in the rhizospheres of different vegetables was assessed by high-throughput sequencing of 16S rDNA amplicons. A total of 3590 and 7532 unique OTUs were identified in root tissue and soil samples, respectively. The complexity of the bacterial community in both microenvironments was evaluated using the alpha components of species richness (Chao1) and diversity (Shannon index). The Shannon index revealed that the rhizosphere soil samples had higher bacterial richness than rhizosphere root samples, although the difference was much smaller than that of Chao1 (Figure 2). The rhizospheres of *B. chinensis* and *L. sativa* var. *angustana* tended to have greater bacterial richness than those of the other vegetable farms. *Proteobacteria*, *Actinobacteria*, *Acidobacteria*, *Chloroflexi* and *Firmicutes* were the five most abundant phyla in soil samples, accounting for approximately 80% of the total OTUs (Figure 3). *Proteobacteria*, *Actinobacteria*, *Bacteroidetes*, *Firmicutes* and *Myxococcota* were the five most abundant phyla in the root samples, accounting for approximately 98% of the total OTUs (Figure 3). *Proteobacteria* were the most dominant bacteria in all samples, accounting for 36.6 ± 8.0% and 78.1 ± 10.9% of the bacterial community in soil and root samples, respectively.

Based on individual phyla in the rhizosphere soil, *Proteobacteria* was the most abundant phyla in soil samples from the *S. oleracea* farm, accounting for 53.7% of the total relative abundance, followed by that in soil samples from *C. bursa-pastoris* (45.6%) and *L. sativa* var. *angustana* (41.2%) farms. The abundance of *Actinobacteria* was the highest in soil samples from the *C. sativum* farm (24.6% of the total relative abundance), followed by that in soil samples from the *B. juncea* farm (21.6%), and then the *A. graveolens* farm (20.9%). The abundance of *Acidobacteria* was the highest in soil samples from the *L. sativa* var. *ramosa Hort*. farm (24.9% of the total relative abundance), followed by that in soil samples from the *A. graveolens* farm (16.4%), and then the *B. narinosa* (14.2%) farm. The abundance of *Chloroflexi* was the highest in soil samples from the *L. sativa* var. *ramosa Hort*. farm (14.0% of the total relative abundance), followed by that in soil samples from the *C. bursa-pastoris* (13.9%) and *B. juncea* (12.4%) farms. The abundance of *Firmicutes* was the highest in soil samples from the *B. juncea* farm (12.9% of the total relative abundance), followed by that in soil samples from the *B. campestris* (7.2%) farm, and then the *L. sativa* var. *angustana* (6.3%) farm.

With regard to individual phyla in rhizosphere roots, *Proteobacteria* was the most abundant in the rhizosphere roots of *L. sativa* var. *angustana* (89.6% of the total relative abundance), followed by that in *B. chinensis* (86.5%) and *B. pekinensis* (85.9%) roots. The abundance of *Actinobacteria* was the highest in *S. oleracea* roots (38.9% of the total relative abundance), followed by that in *C. bursa-pastoris* (23.8%) roots, and then *A. graveolens* roots (21.6%). The abundance of *Bacteroidetes* was the highest in *B. juncea* roots (6.3% of the total relative abundance), followed by that in *C. bursa-pastoris* roots (5.5%), and then *S. oleracea* roots (4.5%). The abundance of *Firmicutes* was the highest in *B. campestris* roots (2.1% of the total relative abundance), followed by that in *B. chinensis* roots (1.9%), and then *C. bursa-pastoris* roots (1.3%). The abundance of *Myxococcota* was the highest in *A. graveolens* roots (2.4% of the total relative abundance), followed by that in *C. bursa-pastoris* (1.2%) and *B. juncea* (0.6%) roots.

### 3.3. Responses of Microbial Communities in the Rhizosphere to Antibiotics

**Figure 4:**
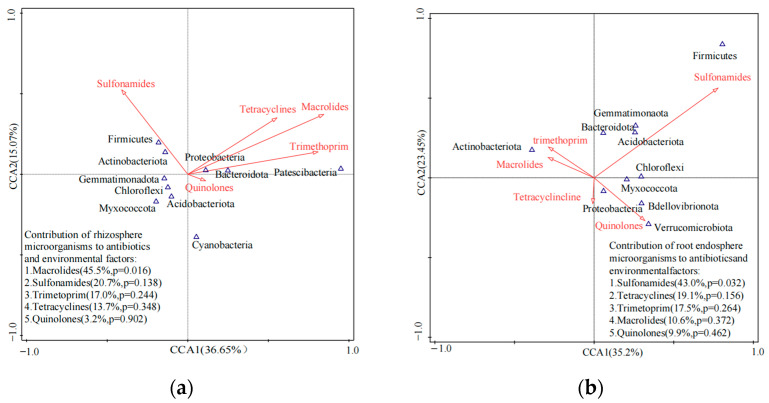
Canonical correlation analysis for microbial community composition (top 10 dominant phyla) and antibiotic groups in rhizosphere samples from different vegetable farms. (**a**) soil samples; (**b**) root samples.

According to the PCoA, soil samples from different vegetable farms exhibited distinct microbial community structures when compared to the root samples (Figure 4). The top 10 dominant phyla in soil samples, including *Proteobacteria*, *Actinobacteria*, *Acidobacteria*, *Chloroflexi*, *Firmicutes*, *Bacteroidetes*, *Gemmatimonadota*, *Cyanobacteria*, *Myxococcota* and *Patescibacteria*, explained 51.7% of the total variation in the first two principal components (Figure 4). The top 10 dominant phyla in root samples (*Proteobacteria*, *Actinobacteria*, *Bacteroidetes*, *Firmicutes*, *Myxococcota*, *Bdellovibrionota*, *Acidobacteria*, *Chloroflexi*, *Gemmatimonadota* and *Verrucomicrobiota*) explained 58.6% of the total variation in the first two principal components (Figure 4). Macrolides in soil samples made the most significant contribution to the results of the study, accounting for 45.5% of the total data variance (*p* < 0.05) and had a positive correlation (R^2^ = 0.655–0.722, *p* < 0.05) with *Proteobacteria*, *Bacteroidetes* and *Patesicibateria*. However, macrolides had a negative correlation with *Chloroflexi* (R^2^ = −0.659, *p* < 0.05). Sulfonamides in root samples made the most significant contribution to the results of the study, accounting for 43.0% of the total data variance (*p* < 0.05), and had a positive correlation (R^2^ = 0.957, *p* < 0.01) with *Firmicutes*, but a negative correlation with *Actinobacteria* (R^2^ = −0.659, *p* < 0.05).

The CCA results showed that residual antibiotics explained 36.7% of the variation in microbial community structure, while microbial community structure explained 2.9% of the variation in residual antibiotics (Table S2 in the Supplementary Material). *Gemmatimonadota* was the most sensitive phylum to antibiotic residuals, with a correlation coefficient of 8.9%, followed by *Actinobacteria*, *Bacteroidetes*, *Patescibacteria*, *Firmicutes*, *Cyanobacteria*, *Myxococcota*, and *Acidobacteria*, which had correlation coefficients greater than 1.0% (Table S2). Macrolide antibiotics were a key factor driving the shifts in the microbial community structure in the soil and had a correlation coefficient of 79.3%. Sulfonamides (42.5%), tetracyclines (40.3%), and trimethoprim (19.8%) were also important factors, while quinolones did not significantly influence microbial community shifts and their correlation coefficients were less than 2.0% (Table S2). The canonical correlation between root microbial community structure and residual antibiotics was relatively low; root microbial community structure explained 17.0% of the variation in residual antibiotics and residual antibiotics explained 5.8% of the variation in root microbial community structure (Table S3 in the Supplementary Material). *Acidobacteria* were the most sensitive phylum to antibiotic residuals, with a correlation coefficient of 38.0%, followed by *Gemmatimonadota*, *Myxococcota*, *Verrucomicrobiota, Chloroflexi*, and *Bdellovibrionota*, which had correlation coefficients greater than 10.0% (Table S3). Macrolide antibiotics were the major factor driving the shifts in the microbial community structure in root tissues, with a correlation coefficient of 14.3%, which was followed by quinolones (11.6%), and then trimethoprim (2.8%). Tetracyclines and sulfonamides did not significantly influence microbial community shifts, and their correlation coefficients were less than 1.0%.

**Figure 5:**
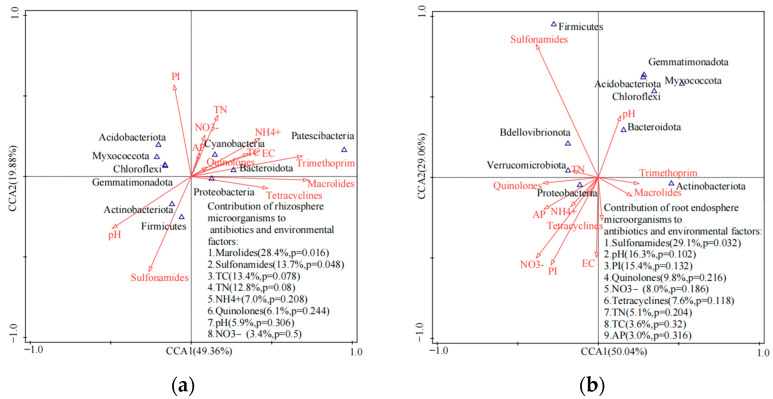
Canonical correlation analysis of microbial community composition (top 10 dominant phyla), antibiotic groups, heavy metals, and nutrient contents in rhizosphere samples from different vegetable farms. (**a**) soil samples; (**b**) root samples. EC is electrical conductivity; AP is available phosphorus; TC is total carbon content; TN is total nitrogen content; NH_4_^+^ stands for soil ammonium nitrogen concentration; NO_3_^−^ represents the soil nitrate nitrogen concentration; PI is a comprehensive index of the analyzed heavy metals (Zn, Cr, Cu, Pb and Ni) and is calculated by the method of Kowalska et al. [33].

Another key factor that contributed to the variations in microbial communities between rhizosphere soil and roots in the studied vegetable farms was the physicochemical properties of the soil (Figure 5). Soil properties, especially nutrients (e.g., TC and TN) were strongly correlated with the proportions of microbial groups in the soil samples. Out of the various physicochemical parameters combined with antibiotic groups, TC was the most significant in determining microbial community structure in soils, with a correlation coefficient of 13.4% (*p* = 0.078). Subsequently, TN (12.8%, *p* = 0.08), NH_4_^+^-N (7.0%, *p* = 0.208), pH (5.9%, *p* = 0.208) and NO_3_^−^-N (3.4%, *p* = 0.50) were also found to be important (Figure 5a). In root samples, pH values were the most significant soil physicochemical parameters influencing microbial communities, with a correlation coefficient of 16.3% (*p* = 0.102), followed by PI (15.4%, *p* = 0.132), NO_3_^−^-N (8.0%, *p* = 0.186), TN (5.1%, *p* = 0.204), TC (3.6%, *p* = 0.32) and AP (3.0%, *p* = 0.316) (Figure 5b). However, other environmental factors, such as pH and heavy metals, were more strongly correlated with the proportions of microbial groups in the root samples (Figure 5).

## 4. Discussion

Numerous antibiotics produced globally are used to prevent diseases or promote growth in livestock and poultry, resulting in substantial amounts of residues in manure and excrement [1,2]. The application of animal and poultry manure to agricultural soils has resulted in the continual entry of antibiotics and antibiotic resistance genes into farmland ecosystems, which has become one of the hotspots of ecological risks posed to agricultural environments [34,35]. The levels of antibiotics, such as tetracyclines, quinolones, sulfonamides, and macrolides in agricultural soils near feedlots, generally range from 10 to 1000 μg/kg, with considerably high concentrations being observed in soils treated with manure-based fertilizers [36,37,38,39]. In the present study, quinolone and tetracycline antibiotics were the dominant contaminants in the soil and root samples analyzed, with high concentrations and detection frequencies being observed. A similar trend of antibiotic residue occurrence was observed in vegetable farms fertilized with manure in the north [34,40], south [41], west [42], and east China [43,44]. The level of antibiotic contamination in soil samples analyzed in this study is equivalent to the median concentrations of residual antibiotics detected in soils fertilized with antibiotic-contaminated manures based on studies conducted in various countries, such as in China, Brazil, the Netherlands, the USA, and Turkey [45]. The concentrations of tetracycline antibiotics (ND-5.3 ng/g) were lower than those reported for organic vegetable farms in China [40,46], Malaysia [46], and Spain [47], and the main compounds detected were chlortetracycline and doxycycline. The concentrations of quinolone antibiotics (ND-27.1 ng/g) were similar to those reported in China [34,45], Brazil [48], and Kenya [49], and the main compounds detected were ofloxacin and enrofloxacin. The concentrations of the remaining sulfonamides, except for sulfamonomethoxine, sulfadiazine, and sulfathiazole, were not detected in either root or soil samples; sulfacetamide had a maximum concentration of 14.4 ng/g. Trimethoprim had a maximum concentration of 36.7 ng/g, which is comparable to the median concentration of 27.93 ng/g reported in meta-analyzed results of studies conducted globally [45]. The levels of macrolides were equivalent to those reported for soils from China, Malaysia, and Brazil [46,48,50]. The concentrations of other antibiotics, such as lincomycin and thiamphenicol, were lower than those in the manure-amended soils [45]. The antibiotic residual patterns of the vegetable soils were consistent with the manure-fertilizers applied in this studied area, implying that the release of antibiotics from manure used as fertilizers is primarily associated with the widespread distribution and persistence of antibiotics in agricultural soils [51]. Consequently, the concentrations of antibiotics vary considerably in soils in different areas, which could be associated with the methods of tillage and fertilization [38,52].

According to the results of this study, the consistent bacterial phyla in the rhizosphere soils were dominated by *Proteobacteria*, *Acidobacteria*, *Actinobacteria*, *Chloroflexi* and *Firmicutes*, which were also frequently observed in other vegetable soils [53,54]. The variations in the dominant bacteria (i.e., relative abundance) in the sampled vegetable fields suggest that *Proteobacteria*, *Acidobacteria*, *Actinobacteria*, *Chloroflexi* and *Firmicutes* are likely to be the functionally active soil microbiomes in these planting systems. It has been demonstrated in prior studies that the same type of bacteria can be observed in different plant species, suggesting that the functional capabilities of these microbiomes can significantly affect the structural and functional diversity of the microbial community [53,54]. Based on the remaining microbial sequences in the rhizosphere microenvironment, the diversity of microbial communities in the roots was lower than that in the soil. Observations similar to this have been made in other plants, including Populus, Arabidopsis and Lactuca [55,56], because only a limited number of microbial species can overcome the plant immune system and establish themselves in the plant [57,58]. The two most abundant phyla in the vegetable roots were *Proteobacteria* and *Actinobacteria*, which can promote plant growth and produce secondary metabolites with antimicrobial properties [56]. The dominant bacterial phyla in vegetable roots were observed to be significantly grown in experiments involving veterinary antibiotics exposure, which are known to contain bacteria with tolerance and degradation capabilities [16,17,18]. In addition, *Bacteroidetes* were abundant in the vegetable roots, which facilitates the degradation of macromolecules, such as starch, cellulose, or proteins [55,56]. *Bacteroidetes* are known to possess antibiotic resistance genes, and are often observed to be forming communities that are resistant to antibiotics after a prolonged period of antibiotic treatment [19,20].

Numerous studies have shown that antibiotics can modify the dominant microbial community in terms of composition, diversity, and richness after being introduced into the soil environment [59,60]. The significant correlations observed between macrolide antibiotics and the dominant phyla in rhizosphere soils, as well as sulfonamide antibiotics in rhizosphere roots demonstrated the considerable impact of antibiotic residues on microbial communities in the present study. The results showed that macrolide and sulfonamide antibiotics significantly influenced the microbial community structure, although their residual concentrations in the vegetable soils were substantially low, which is consistent with the results of previous studies conducted on aquaculture sediment and farmland soils in natural settings [9,10,61]. Macrolide antibiotics, which have broad-spectrum activity against many Gram-positive bacteria, can significantly decrease microbial diversity [62]. The strong correlation observed between the presence of macrolide antibiotics and certain bacterial phyla (e.g., *Proteobacteria*, *Acidobacteria*, *Actinobacteria*, *Firmicutes*, and *Chloroflexi*) in this study may be due to potential macrolide-resistant bacteria using macrolides as carbon and energy sources. Sulfonamides are known to inhibit bacterial growth by altering the microbial production of folic acid, which can in turn, alter the microbial community structure and diversity at concentrations currently present in the environment [63]. No significant correlation was observed between the dominant occurrence of quinolone and tetracycline antibiotics and microbial community composition in this study. The adsorption of these antibiotic groups onto the mineral phase of soil is a key factor influencing their mobility, stabilization, bioavailability, and bioaccessibility to microorganisms [64,65]. A bacterial community may develop antibiotic tolerance, which could also account for the lack of observed effects [66,67].

**Figure 6:**
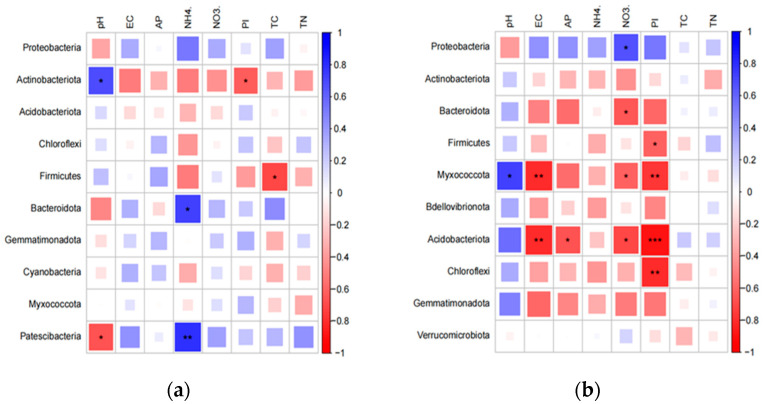
A heatmap of microbial community composition (top 10 dominant phyla) and soil properties in two rhizosphere microenvironments; (**a**) soil samples; (**b**) root samples. EC is electrical conductivity; AP is available phosphorus; TC is total carbon content; TN is total nitrogen content; NH_4_^+^ stands for soil ammonium nitrogen concentration; NO_3_^−^ represents the soil nitrate nitrogen concentration; PI is a comprehensive index of the analyzed heavy metals (Zn, Cr, Cu, Pb and Ni) and is calculated by the method of Kowalska et al. [33]; * denotes significant differences (*p* < 0.05) between the relationships, ** denotes significant difference (*p* < 0.01), and *** denotes significant difference (*p* < 0.001).

The interaction between antibiotics and soil matrices, such as organic matter, may reduce biological activities of antibacterial molecules before they reach bacteria [68,69]. The observed lack of effect of antibiotic residues, such as quinolone and tetracycline antibiotics on microbial community composition in this study may be due to the physicochemical properties of the antibiotics, such as sequestration and low bioavailability [63]. The potent antimicrobial effects of antibiotics in soils can differentially inhibit the growth of soil microorganisms, and consequently, influence the soil microbial community composition, which may result from the inhibition of microbial growth by different soil properties to a greater or lesser extent [70,71]. Soil properties are key factors shaping the composition of microbial communities [9,10,61]. According to previous findings, soil properties tend to shape microbial diversity in environments where antibiotic residues are present. In particular, TC and pH have a negative correlation with *Sulfurovum*, *Sulfurimonas*, and *Desulfobulbus*, but a positive correlation with *Methylophaga* in aquaculture farms with long-term antibiotic application [15]. The interactions between plant-associated microbial communities can be affected by several environmental factors, such as nutrient availability, bulk density, and soil pore size [56,58]. The structure of microbial communities in soils surrounding the roots is shaped by the soil characteristics, and the composition and abundance of the root exudates [56,58]. Therefore, soil physicochemical properties play a key role in shaping microbial communities in the rhizosphere (Figure 6). Because microbes in the roots are largely recruited from the rhizosphere, it is expected that factors affecting the microbial community in the rhizosphere will also affect the microbial community in the roots, and the increased level of antibiotic residues in agricultural soils due to the use of manure-based fertilizers poses a health risk through the migration and diffusion of antibiotics in agricultural ecosystems [5,6,7,8]. However, the mechanisms underlying the effect of antibiotic residues on microbial communities surrounding and in the roots of food crops remain unclear. Therefore, information regarding the effects of antibiotic residues on microbial communities is essential for their effective management; however, antibiotic residues pose a major challenge due to the interactions between antibiotics and environmental factors.

## 5. Conclusions

Our study revealed that antibiotic residues considerably shifted the microbial community composition in rhizosphere soils and roots in various vegetable farms. The most prevalent antibiotics were quinolones and tetracyclines, with individual compounds having a maximum concentration of 27.1 ng/g. Macrolides and sulfonamides were less prevalent and had low detection frequencies. The five most abundant phyla in soil samples were *Proteobacteria*, *Actinobacteria*, *Acidobacteria*, *Chloroflexi*, and *Firmicutes*, while the five most abundant phyla in root samples were *Proteobacteria*, *Actinobacteria*, *Bacteroidetes*, *Firmicutes*, and *Myxococcota*. Macrolides and sulfonamides significantly contributed to the variations in microbial community composition, in addition to soil properties, such as TC, TN, and pH. However, TC and TN were more closely associated with the proportions of microbial communities in soil samples, while other environmental factors, such as pH and heavy metal levels, were more closely associated with the microbial communities in root samples. Therefore, this study provides essential information regarding the effects of antibiotic residues on microbial communities and their interactions with environmental factors.

## Figures and Tables

**Table 1:** Basic physical and chemical properties of rhizosphere soil.

**pH**	**EC**	**AP**	**TC**	**TN**	**NH_4_^+^**	**NO_3_^−^**
6.9 ± 0.4	553.4 ± 461.2	79.5 ± 39.6	16,990 ± 6210	3360 ± 1650	4.4 ± 0.6	80.6 ± 82.3
**As**	**Zn**	**Cr**	**Cu**	**Pb**	**Cd**	**Ni**
16.17 ± 4.0	330.8 ± 190.9	41.22 ± 13.07	20.3 ± 16.0	56.6 ± 21.5	0.4 ± 0.2	9.1 ± 3.5

EC is electrical conductivity, μS/cm; AP is available phosphorus; TC is total carbon content; TN is total nitrogen content; except EC/pH, all units in the table are mg/kg.

## Data Availability

Not applicable.

## Supplementary Materials

The following Supplementary Materials can be downloaded at: https://www.mdpi.com/article/10.3390/ijerph20043137/s1, Table S1. Multiple reaction monitoring parameters of the antibiotics analyzed; Table S2. Canonical correlation-redundancy analysis between microbial community structure (dominant phyla) and residual antibiotics in rhizosphere soil samples from different vegetable farms; Table S3. Canonical correlation-redundancy analysis between microbial community structure (dominant phyla) in root tissues and residual antibiotics in rhizosphere soil samples from different vegetable farms.
