# Supplementary material for: Adaptation of Rhizosphere Microbial Communities to Continuous Exposure to Multiple Residual Antibiotics in Vegetable Farms
Source: Int J Environ Res Public Health. 2023 Feb 10;20(4):3137. doi: 10.3390/ijerph20043137 (PMC9958589; doi:10.3390/ijerph20043137)
Supplement: Supplementary file 1 [file ijerph-20-03137-s001.zip › ijerph-2086832-supplementary.pdf]

Supplementary Material

Adaptation of Rhizosphere Microbial Communities to Continuous Exposure to Multiple Residual Antibiotics in Vegetable Farms

Jincai Qiu[1], Yongshan Chen[2]*, Ying Feng[2], Xiaofeng Li[2], jinghua Xu[2], Jinping Jiang[3]

[1]School of advanced manufacturing, Fuzhou University, 362200, Quanzhou, China
[2]School of Resources and Environmental Science, Quanzhou Normal University, 362000, Quanzhou, China.
[3]Guangxi Scientific Experiment Center of Mining, Metallurgy and Environment, Guilin University of Technology, 541004, Guilin, China.

*Corresponding author. Email: yshchen421@163.com

Table S1 Multiple reaction monitoring parameters of the antibiotics analyzed

Antibiotics		Precursor ion (m/z)	Product ions (m/z)	
Tetracyclines	Doxycycline	445.2	428.2	154
	Tetracycline	445.3	154	410.2
	Oxytetracycline	461.2		443.1
	Chlortetracycline	479.3	444.2	462.2
Sulfonamides	Sulfadiazine	251	92	156
	Sulfamonomethoxine	281	92	156
	Sulfamethoxazole	254	92	156
	Sulfacetamide	215	156	108
	Sulfadimethoxine	311.1	92	156
	Sulfamethazine	279.1	92	186
	Sulfaquinoxaline	301.1	92.2	156.1
	Sulfathiazole	256	92	156
Quinolones	Ofloxacin	362.1	261.1	318.1
	Norfloxacin	320.1	233	276.1
	Ciprofloxacin	332.1	314.1	288.1
	Enrofloxacin	360.2	316.1	245
	Lomefloxacin	352.1	265.1	308.1
	Difloxacin	400.2	356.1	299
Macrolides	Erythromycin	734.4	158.1	576.3
	Roxithromycin	837.5	158	/
	Azithromycin	749.9	157.8	591.4
	Tylosin	916.5	174.1	101.1
Chloramphenicol	Thiamphenicol	356.296	299.26	279.216
Lincosamides	Lincomycin	407.36	126.19	359.422
Trimethoprim	Trimethoprim	291	230	123

Table S2. Canonical correlation-redundancy analysis between microbial community structure (dominant phyla) and residual antibiotics in rhizosphere soil samples from different vegetable farms.

Standardized variance of the antibiotics explained by		Canonical correlation R2 between the two first canonical variables	Standardized variance of microbial community structure (dominant phyla)	
microbial community structure (dominant phyla)			Explained by Residual antibiotics	
0.0293			0.3669	
Squared multiple correlations between Microbial Community Structure and the first M canonical variables of Residual Antibiotics			Squared multiple correlations between Residual Antibiotics and the first M canonical variables of Microbial Community Structure	
M	1		M	1
Gemmatimonadota	0.0894		Macrolides	0.7929
Actinobacteriota	0.0566		Sulfonamides	0.4245
Bacteroidota	0.0423		Tetracyclines	0.4028
Patescibacteria	0.0394		Trimethoprim	0.1982
Firmicutes	0.0033		Quinolones	0.0162
Cyanobacteria	0.0229			
Myxococcota	0.0240			
Acidobacteriota	0.0151			
Proteobacteria	0.0000			
Chloroflexi	0.0000			

Table S3. Canonical correlation-redundancy analysis between microbial community structure (dominant phyla) in root tissues and residual antibiotics in rhizosphere soil samples from different vegetable farms.

Standardized variance of the antibiotics explained by		Canonical correlation R2 between the two first canonical variables	Standardized variance of microbial community structure (dominant phyla)	
microbial community structure (dominant phyla)			Explained by Residual antibiotics	
0.0583			0.1699	
Squared multiple correlations between Microbial Community Structure and the first M canonical variables of Residual Antibiotics			Squared multiple correlations between Residual Antibiotics and the first M canonical variables of Microbial Community Structure	
M	1		M	1
Acidobacteriota	0.3802		Macrolides	0.1430
Gemmatimonadota	0.3527		Quinolones	0.1158
Myxococcota	0.3053		Trimethoprim	0.0280
Verrucomicrobiota	0.2593		Tetracyclines	0.0047
Chloroflexi	0.2090		Sulfonamides	0.0009
Bdellovibrionota	0.1373			
Actinobacteriota	0.0259			
Bacteroidota	0.0171			
Firmicutes	0.0131			
Proteobacteria	0.0000			
